# Psychosocial adaptation of siblings of autistic individuals: current evidence, controversies, and future directions

**DOI:** 10.3389/fpsyg.2026.1858545

**Published:** 2026-09-03

**Authors:** Yaqi Feng, Jingxia Zhang, Rong Zhang

**Affiliations:** Department of Child and Adolescent Mental Health, The Second Hospital of Gansu Province, Lanzhou, Gansu, China

**Keywords:** autism spectrum disorder, family functioning, family-centered care, pediatric psychology, psychosocial adaptation, resilience, siblings

## Abstract

This mini review synthesizes current evidence on the psychosocial adaptation of non-autistic siblings of autistic children, adolescents, and adults. We searched PubMed, PsycINFO, and Web of Science (January 2021–March 2026) and synthesized the retrieved literature narratively along three organizing axes: ecological level, developmental stage, and the risk–resilience continuum. Existing research shows a heterogeneous profile in which vulnerability and resilience coexist: siblings may experience loneliness, anxiety, depressive symptoms, and role strain, but may also report empathy, maturity, and a strong sense of responsibility, alongside acceptance and benefit-finding. Adaptive outcomes are associated with multiple interacting factors, including the severity of co-occurring behavioral and emotional difficulties in the autistic child, sibling age and sex, parental mental health, family communication, social support, school context, and the interacting influences of gender, religiosity, and culture. Emerging interventions, particularly psychoeducational support groups, play-based sibling training, and family-oriented programs, have established feasibility, acceptability, and social validity, and a single randomized trial supports improved sibling relationship quality; established effectiveness has not yet been demonstrated. Most primary studies are cross-sectional, qualitative, or small-scale, and a substantial proportion draws on mixed neurodevelopmental, intellectual-disability, or chronic-condition samples, so autism-specific inference remains limited. Current controversies concern how to balance risk-based and strength-based framings without pathologizing ordinary ambivalence or romanticizing caregiving-related growth, how far elevated sibling risk reflects shared familial liability rather than psychosocial burden, and how cultural context reshapes sibling roles and the meaning of adaptation. Future research should prioritize longitudinal designs, culturally responsive interventions, and greater attention to preschool-aged and adult siblings to better inform family-centered pediatric psychological care.

## 1 Introduction

Autism spectrum disorder (ASD) is a neurodevelopmental condition, typically identified in early childhood, involving differences in social communication and social interaction together with restricted, repetitive patterns of behavior, interests, or activities, and associated with a wide range of support needs (American Psychiatric Association, 2022). Because those support needs are distributed across the family system, they shape the everyday lives of parents and siblings as well as of the autistic person (Quatrosi et al., 2023); non-autistic siblings, in particular, share the longest cohabitation and developmental trajectory with the autistic individual. Clinical and research attention has focused predominantly on the autistic individual and their parents; with the growing influence of family systems theory and ecological developmental perspectives, however, researchers increasingly recognize that having an autistic brother or sister represents both a potential source of adversity and a context for personal growth (Watson et al., 2021; Schmeer et al., 2021). This population may encounter emotional distress, behavioral difficulties, reduced quality of life, academic difficulties, and complex sibling dynamics (Godara et al., 2024; Huang et al., 2022); they may equally demonstrate resilience, empathy, and a heightened sense of responsibility (Mokoena and Kern, 2022; Oti-Boadi et al., 2025).

A note on terminology is warranted. We use identity-first language, which is preferred by many autistic people and self-advocacy organizations, and refer throughout to “non-autistic siblings.” We deliberately avoid “unaffected siblings,” which is inconsistent with the very evidence reviewed here, and “typically developing siblings,” which obscures the fact that some siblings themselves have neurodevelopmental characteristics or diagnoses. Where a primary study used different terminology or sampled a broader population, we describe that population as the original authors defined it.

Rather than re-cataloging this literature, this Mini Review uses three organizing axes—ecological level (individual, family, and environment), developmental stage (infancy and preschool, school age and adolescence, and adulthood), and the risk–resilience continuum—as a heuristic for arranging and comparing evidence. We present these axes as an organizing device rather than as a formal analytic model, and we return to them explicitly at the close of each section; the evidence base is at present too uneven, particularly at the preschool and adult ends of the developmental axis, to support a symmetrical treatment of every cell. Throughout, we signal the design and population of the underlying evidence and state whether a finding is autism-specific or is best read as convergent evidence from the wider intellectual and developmental disability (IDD) literature.

## 2 Approach to literature

Consistent with the Mini Review format, this is a structured but narrative synthesis rather than a systematic review. It was not prospectively registered and does not follow PRISMA guidance.

Sources and search terms. PubMed, PsycINFO, and Web of Science were searched from January 2021 to March 2026, with earlier landmark papers added by hand-searching reference lists of retrieved reviews. Three concept blocks were combined with AND—sibling terms, autism terms, and psychosocial, adjustment, and intervention terms—with the full search strings for each database given in Supplementary material S1.

Eligibility, selection, and synthesis. We included peer-reviewed empirical studies and reviews in English reporting outcomes, experiences, or interventions concerning siblings of autistic individuals; studies of siblings of individuals with IDD, Down syndrome, or other chronic conditions were included only where autism-specific evidence was sparse and are labeled as such throughout. We excluded conference abstracts, dissertations, editorials, and studies reporting outcomes of the autistic individual alone, except where cited to illustrate bidirectional processes. Titles and abstracts were screened by one author and checked by a second, with disagreements resolved by discussion; included records were charted by population, developmental stage, design, autism specificity, and principal finding (Table 1), then synthesized narratively along the three axes above.

**Table 1:** Representative evidence on psychosocial adaptation, determinants, and support strategies for siblings of autistic individuals.

Theme	Representative studies	Study design	Population and developmental stage	Autism-specific?	Principal findings	Main limitations of the evidence
Overall psychosocial profile	Quatrosi et al. (2023), Watson et al. (2021)	Scoping review; systematic review of heterogeneous primary studies	Siblings from childhood to adulthood	Yes (autism-focused reviews)	Adaptation is heterogeneous: some siblings report poorer quality of life, loneliness, anxiety, or depressive symptoms, whereas others report empathy, maturity, and growth. A dual risk–resilience framing is preferable to either alone.	Pooled across ages and designs; underlying primary studies predominantly cross-sectional or qualitative; no meta-analytic estimate.
Mental health and life-course risk	Lin et al. (2022), Sipowicz et al. (2022), Wolff et al. (2023), and Shtayermman and Fletcher (2022)	National register-based cohort; cross-sectional surveys	Children to adults; siblings and, in Shtayermman and Fletcher (2022), autistic individuals combined	Partly (Wolff et al. (2023) covers neurodevelopmental conditions broadly; Shtayermman and Fletcher (2022) combines probands and siblings)	Signals of higher rates of developmental and mental disorders, loneliness, depression, and suicide-related outcomes among siblings, supporting routine attention to sibling wellbeing.	Register data cannot separate shared familial liability from psychosocial burden; suicide-related findings come from small cross-sectional samples without sibling-specific estimates; no causal inference.
Sibling relationship quality	Hayden et al. (2023), Brown et al. (2025), Rixon et al. (2022), and Rixon et al. (2021)	Cross-sectional observational studies; latent-profile analysis	School-age children and adolescents	Partly (Hayden et al. (2023) intellectual disability; Rixon et al. (2022) and Rixon et al. (2021) autism with intellectual disability)	Relationship quality is bidirectional and ranges from positive to low-involvement or conflictual; positive profiles co-occur with better family and peer functioning.	Cross-sectional; profiles describe co-occurrence, not developmental sequence; partly non-autism-specific samples.
Child characteristics and severity	Fredriksen et al. (2025), Rixon et al. (2022), and Rixon et al. (2021)	Observational predictive and subgroup (latent-profile) analyses	School-age siblings	Partly (chronic-disorder and autism-plus-intellectual-disability samples)	Severity, particularly externalizing behavior and anxiety in the autistic child, is more strongly associated with sibling maladjustment and conflict than diagnostic category alone.	Small subgroups; mixed diagnostic samples; associations, not effects.
Family and social support	Koukouriki et al. (2022), Koukouriki et al. (2021), Chang et al. (2024), Brinkman et al. (2023), Hamama (2024), and Schumann et al. (2024)	Cross-sectional survey; qualitative thematic analysis of parent–sibling dialogs	Mainly mothers and school-age siblings; mostly Greek, Israeli, Taiwanese, and US samples	Partly (Schumann et al. (2024); chronic disorders generally)	Parental anxiety, overcontrol, affiliate stigma, and a negative emotional climate accompany poorer sibling outcomes, whereas direct communication, validation, and perceived social support buffer loneliness and distress.	Reliance on parent report and shared-informant variance; cross-sectional; limited cultural range; one key analysis not autism-specific.
School, culture, gender, and religiosity	Pavlopoulou et al. (2022), Long et al. (2022); Alon (2025), and Alon (2026)	Qualitative interview studies; cross-sectional comparative surveys	School-age children to emerging adults	Partly (Alon (2025) and Alon (2026) combine autism with Down syndrome and cerebral palsy)	School understanding and cultural role expectations shape how siblings interpret responsibility, identity, and future obligations; gender, religiosity, and diagnosis interact rather than add, with the highest stress among siblings of autistic individuals in more traditional communities.	Small purposive qualitative samples; cross-sectional; cross-cultural measurement invariance untested; limited generalisability.
Psychoeducational and group interventions	Zucker et al. (2022), Kuhlthau et al. (2023), Fell et al. (2022), and Kaur et al. (2026)	One randomized controlled trial; one randomized pilot trial; qualitative acceptability study; scoping review	School-age and adolescent siblings	Yes	Efficacy for improved sibling relationship quality is supported by a single randomized trial; feasibility, acceptability, and social validity are established for virtual and in-person group formats.	One trial with short follow-up and no replication; pilot not powered for efficacy; positive trends should not be reported as treatment effects.
Behavioral and play-based interventions	Watkins et al. (2021) and Glugatch and Machalicek (2021)	Single-case experimental designs	Child siblings	Yes	Play-based sibling training increases sibling-initiated interaction and reciprocal play, with generalization and maintenance; social validity endorsed by parents and siblings.	Very small samples; experimental control demonstrated within individuals only; no average effect estimates; population-level effectiveness not established.
Infant-sibling early intervention (conceptually distinct literature)	Riva et al. (2021) and Annunziata et al. (2024)	Pilot study; controlled trial (ongoing)	Infant siblings at elevated likelihood of autism	Yes, but a different target population and aim	Early intervention may improve social communication and language in infant siblings; stratified ecological monitoring is under evaluation.	Targets the infants’ own emerging development, not psychosocial adaptation in the sibling role; findings are not transferable to the questions reviewed here.
Major gaps and priorities	Prentice et al. (2025), Burke et al. (2024), Long et al. (2025), Lemoine and Schneider (2025), and Stelter et al. (2026)	Scoping reviews; cross-sectional studies; program development	Preschool-aged and adult siblings (both under-represented)	Partly (much of the adult evidence is from intellectual and developmental disability or Down syndrome samples)	Preschool and adult siblings, culturally adapted programs, future-planning needs, and longitudinal or adequately powered randomized studies remain the principal priorities.	Evidence sparse at both ends of the developmental axis; adult-sibling literature largely not autism-specific.

“Autism-specific” indicates that the study sample consisted exclusively of siblings of autistic individuals. Study design and limitations are reported descriptively; no formal quality appraisal or certainty grading (e.g., GRADE) was undertaken. Bracketed numbers correspond to the reference list.

Limitations of this approach. No formal risk-of-bias assessment or certainty grading was undertaken, study selection was purposive rather than exhaustive, and the restriction to English-language sources probably under-represents research from non-English-speaking regions. Selection and language bias therefore cannot be excluded, and this synthesis should be read as an interpretive map of a fast-moving field rather than an exhaustive account of it.

## 3 Psychosocial adaptation: risk, resilience, and bidirectional effects

The psychosocial adaptation of non-autistic siblings presents a complex and heterogeneous picture, in which risk and resilience coexist and the influence of sibling relationships operates bidirectionally. Because the evidence below derives largely from school-aged and adolescent samples and is predominantly cross-sectional, the associations described should not be read as causal effects, nor generalized uncritically to preschool-aged or adult siblings.

Cross-sectional comparisons indicate elevated psychosocial risk. Compared with controls, siblings of autistic children report higher loneliness, social dissatisfaction, anxiety, and depressive symptoms (Sipowicz et al., 2022; Koukouriki et al., 2022; Koukouriki et al., 2021). A nationwide register-based cohort study reported that siblings of autistic individuals had an increased likelihood of subsequently receiving diagnoses of developmental delay, speech and language disorders, attention-deficit/hyperactivity disorder, anxiety disorders, and disruptive behavioral disorders relative to controls (Lin et al., 2022). Such register findings are, however, open to a competing interpretation central to this literature: because siblings share genetic and environmental liability with the autistic proband, elevated diagnostic rates may reflect familial aggregation of neurodevelopmental traits rather than the psychosocial consequences of growing up with an autistic brother or sister. Register data cannot separate these pathways, and reading them as evidence of caregiving burden overstates what the design supports.

Findings on suicide-related outcomes warrant particular caution. One small cross-sectional study identified anxiety and sexual orientation as correlates of suicide attempts in a combined sample of autistic individuals and their siblings, without reporting sibling-specific estimates (Shtayermman and Fletcher, 2022); a separate cross-sectional study of siblings of people with a range of neurodevelopmental conditions, not autism alone, reported higher rates of non-suicidal self-injury, suicidal ideation, and lifetime suicide attempts than controls (Wolff et al., 2023). These are hypothesis-generating signals, not estimates of prevalence, autism specificity, or causation.

However, to pathologize the sibling experience entirely would be reductive. Many siblings describe exceptional empathy, patience, and a sense of responsibility (Schmeer et al., 2021; Mokoena and Kern, 2022), and describe themselves as caregivers and protectors whose relationship has shaped their identity and career trajectory (Mokoena and Kern, 2022; Viswanathan et al., 2022), and qualitative work shows families sustaining closeness through shared routines (Trew, 2025a). These are participant-reported experiences rather than measured outcomes, and they coexist with, rather than cancel out, the distress described above.

Beyond cataloging positive traits, recent work points to psychological processes that organize resilient adaptation. Acceptance of the brother or sister is emerging as a pivotal construct, shaping both the sibling bond and willingness to provide future care; in a large cross-sectional study of emerging-adult siblings of individuals with Down syndrome or autism, optimism and emotions held toward the sibling predicted acceptance (Alon, 2026). Because that sample combined Down syndrome and autism, the finding is convergent rather than autism-specific. Many siblings likewise describe benefit-finding alongside genuine ambivalence, holding warmth, loyalty, worry, and frustration simultaneously. Reframing resilience in terms of acceptance, meaning-making, and the management of ambivalence, rather than the presence of empathy or maturity, helps explain why siblings in comparable circumstances follow markedly different trajectories.

The sibling relationship is itself bidirectional. Externalizing behavior and anxiety in the autistic child are associated with greater sibling conflict (Rixon et al., 2022; Rixon et al., 2021); conversely, the prosocial, internalizing, and externalizing behavior of the non-autistic sibling is associated with closeness–companionship and conflict–rivalry in a study of children with intellectual disability, only a subset of whom were autistic (Hayden et al., 2023). Latent profile analysis has classified sibling relationships of autistic youth as positive, negative, or low-involvement, with the majority (64.5%) low-involvement and positive relationships clustering with adaptive family and peer functioning (Brown et al., 2025). Because these profiles are cross-sectional, the clustering describes co-occurrence rather than developmental sequence; and because several key relational findings derive from intellectual-disability or mixed neurodevelopmental samples, in which behavioral profiles, stigma, and caregiving demands differ, they are best read as convergent rather than strictly autism-specific.

## 4 Key factors influencing sibling psychosocial adaptation

The psychosocial adaptive outcomes of siblings of autistic children are shaped by multiple factors operating across individual, familial, and broader environmental levels in an interrelated manner, and the relative salience of these factors shifts across the developmental axis.

At the individual level, the characteristics of the autistic child are a primary correlate. In an observational study of siblings of children with a range of chronic disorders, only some of whom were autistic, condition severity was more strongly associated with sibling mental health than diagnostic category was (Fredriksen et al., 2025). Externalizing (e.g., aggression) and internalizing (e.g., anxiety) problems in the autistic child are likewise associated with greater difficulties and relationship conflict in the non-autistic sibling (Rixon et al., 2022; Rixon et al., 2021; Hayden et al., 2023). Sibling characteristics also matter, although less consistently than is often assumed. Younger siblings may experience greater confusion due to limited understanding (Fjermestad and Lervik, 2025), whereas older siblings, particularly sisters, more often assume caregiving responsibilities, with attendant role ambiguity and stress (Trew, 2025b). In an emerging-adult sample, female siblings reported higher negative affect and family cohesion, and sex moderated the association between family cohesion and positive affect (Hamama and Gaber, 2021). Evidence on birth order is mixed and has more often been examined in relation to the autistic child’s own development than to sibling adjustment (Koukouriki et al., 2022; McFayden et al., 2022). Increasingly, psychological resources rather than demographics alone are treated as proximal determinants: dispositional optimism and the affective stance a sibling holds toward their brother or sister jointly predict acceptance, an association that appears especially marked for brothers (Alon, 2026). This reframes acceptance and optimism as potentially modifiable rather than fixed, although the cross-sectional design and mixed sample leave both the direction of influence and the autism specificity unresolved.

Family factors are the central systemic context. Parental mental health and parenting matter most: maternal anxiety and depressive symptoms are associated with sibling depressive symptoms (Koukouriki et al., 2021), mothering and mother–child interaction have been reported to differ between autistic children and their non-autistic siblings (Chang et al., 2024), and parental emotional expressiveness and affiliate stigma have been linked to sibling internalizing behavior (Brinkman et al., 2023). These associations rest largely on cross-sectional and parent-reported data, and shared-informant variance may inflate them. Dedicated parental time and age-appropriate education about autism are described by siblings as protective (Romney et al., 2025), whereas restricted family activities, concerns about the future, and conflict at home are the principal challenges siblings raise with parents (Schumann et al., 2024), in an analysis of families of children with a range of chronic disorders rather than autism alone. Lower family socioeconomic resources have been associated with poorer sibling quality of life in a single-site cross-sectional study (Arya et al., 2023), a thinly evidenced claim that should not be generalized.

Environmentally, perceived social support, particularly from family, is associated with lower loneliness and higher life satisfaction (Koukouriki et al., 2022; Hamama, 2024), although it may not fully offset the effects of parental anxiety (Koukouriki et al., 2021). At school, limited personal time and disrupted sleep can affect academic performance, and siblings sometimes explain their brother’s or sister’s needs to staff (Pavlopoulou et al., 2022). Cultural context shapes role perceptions and self-concept: Latinx siblings tend to emphasize family roles, positioning themselves as caregivers and protectors, whereas non-Latinx White siblings emphasize sensitivity to individual differences (Long et al., 2022). Religiosity and gender operate within this cultural layer rather than alongside it. In a cross-sectional study of 465 emerging-adult siblings, brothers reported higher stress than sisters, and religious-cultural sector moderated this effect, with the highest stress reported by siblings of autistic individuals in more traditional, less secular communities (Alon, 2025). Religiosity is thus double-edged: religious meaning systems can furnish coping, purpose, and communal support, yet normative expectations around family honor and silence about disability may intensify internalized pressure. Read across the ecological axis, these are not independent moderators but intersecting forces; read across the developmental axis, their weight shifts, with parenting and comprehension dominant in early childhood, peer and school processes in adolescence, and cultural caregiving scripts in emerging adulthood.

## 5 Intervention and support strategies: current models and what they establish

Intervention and support strategies have progressively evolved, but their claims require careful calibration: feasibility, acceptability, social validity, preliminary efficacy, and established effectiveness are distinct thresholds, and most of this literature has reached only the first three.

Psychoeducational support groups are the most prevalent modality, combining autism-related psychoeducation, emotional expression, stress-management skills, and peer support. The strongest single piece of evidence is a randomized controlled trial in which participation in a sibling support group focused on autism and coping skills resulted in significant improvements in sibling relationship quality compared with siblings who attended a structurally similar non-autism-themed group (Zucker et al., 2022). This constitutes a genuine efficacy signal, but it rests on one trial with a short follow-up and no independent replication. A randomized pilot trial of a virtual resilience-based group intervention for adolescents demonstrated good feasibility and acceptability, with participants showing positive trends in stress management and resilience (Kuhlthau et al., 2023); as a pilot, it was not powered to test efficacy, and these trends should not be reported as treatment effects. Participants report that such groups help them understand their siblings’ differences, build peer networks, and acquire coping skills (Kaur et al., 2026; Fell et al., 2022); these are acceptability data rather than outcome data.

Behavioral skills training typically trains non-autistic siblings as “play partners” or “peer tutors” through structured, interest-based play with adult guidance, modeling, and feedback (Watkins et al., 2021), increasing sibling-initiated interactions and reciprocal play with generalization and maintenance over time (Watkins et al., 2021; Glugatch and Machalicek, 2021). These findings derive from single-case experimental designs, which can demonstrate experimental control within individuals but cannot estimate average effects or support claims of population-level effectiveness; in one comprehensive package, the support-group component could not be evaluated, so what was established was social validity endorsed by parents and siblings, not efficacy (Glugatch and Machalicek, 2021).

A conceptually distinct literature targets infant siblings at elevated likelihood of autism; its aim is early identification and remediation of the infants’ own emerging developmental difficulties, not psychosocial support for siblings in the sibling role. A pilot study reported gains in social communication and language (Riva et al., 2021), and a controlled trial is under way (Annunziata et al., 2024). Its findings should not be transferred to the psychosocial questions considered here. For older siblings, interventions promoting open parent–sibling dialog are important: the “SIBS” intervention includes sessions in which siblings voice wishes and challenges while parents learn to listen and validate (Schumann et al., 2024), though it was developed for chronic disorders generally and autism-specific effectiveness data are not yet available.

Most studies remain limited by small samples and heterogeneous protocols, outcomes, and methods, and have focused on school-aged siblings (Godara et al., 2024; Lynam and Smith, 2022); work with preschool-aged (Prentice et al., 2025) and adult (Burke et al., 2024) siblings is scarce, and cultural adaptation is largely unaddressed. Two priorities follow: support should be culturally and religiosity-sensitive rather than uniform, and could target modifiable processes such as optimism and acceptance (Alon, 2025; Alon, 2026); and programs engaging adult siblings in future planning are only beginning to be developed and evaluated (Long et al., 2025; Stelter et al., 2026), mostly outside autism-specific samples.

## 6 Perspectives and controversies: risk, resilience, and their interpretive hazards

This field has been framed primarily through risk and resilience models. These are better understood as ends of a continuum than as competing accounts; the substantive debate concerns the conditions under which each applies and the interpretive hazards each carries. The risk model, grounded in family stress theory, treats caregiving demands and resource constraints as chronic stressors that may deprive siblings of needed attention (Schmeer et al., 2021; Brinkman et al., 2023); the resilience model emphasizes strengths developed through navigating adversity—empathy, responsibility, maturity, and problem-solving (Mokoena and Kern, 2022; Oti-Boadi et al., 2025; Iannuzzi et al., 2022)—and family processes such as routines, communication, and collective coping (Trew, 2025a; Critchley et al., 2021). Neither framing is neutral, and five issues deserve more explicit attention than they usually receive.

First, ordinary ambivalence should not be pathologized. Feeling love, irritation, worry, and embarrassment toward a brother or sister is normative in sibling relationships generally, not a symptom. Instruments designed to detect distress will register such feelings as problems unless normative sibling comparison data are used, and services then risk problematizing family life that is demanding but unremarkable.

Second, and symmetrically, caregiving-related growth should not be romanticized. Reports of maturity are self-reported, gathered from siblings who consent to research and who may draw on culturally available narratives of the “special sibling.” Benefit-finding may reflect genuine development, adaptive meaning-making, or social desirability, and current designs cannot distinguish these; presenting growth as a compensating benefit risks justifying the withdrawal of support.

Third, responsibility and maturity are not uniformly positive. Behaviors described here as early maturity are, in adjacent literatures, termed parentification or role reversal, in which a child’s own developmental needs are subordinated to family caregiving. Whether responsibility functions as competence or as burden appears to depend on its scale, voluntariness, and acknowledgment—questions this field has rarely asked directly.

Fourth, the assumption that siblings will become future caregivers deserves scrutiny. Much clinical and policy discourse treats sibling succession in caregiving as natural and desirable, creating a real tension between family responsibility and sibling autonomy. Adult siblings report that appraisals of the disability’s impact translate into concrete life decisions, including where to live and which career to pursue, with sisters in particular wishing to remain geographically close (Lemoine and Schneider, 2025). Framing future caregiving as an expectation rather than a choice risks constraining self-determination; support programs should make bounded involvement, or non-involvement, a legitimate option.

Fifth, an explanatory rather than ethical issue: siblings share genetic and neurodevelopmental liability with autistic probands, so part of the “sibling risk” documented in register and endophenotype studies (Lin et al., 2022; Seng et al., 2021) may be familial rather than experiential in origin. Distinguishing the two requires designs this literature has largely not used—prospective cohorts measuring siblings’ own neurodevelopmental traits, or comparison with siblings of children with non-heritable chronic conditions. Until then, the causal language common in this area outruns the evidence.

The two models are not mutually exclusive, and positive family functioning and social support accompany resilience where externalizing behavior in the autistic child accompanies risk (Rixon et al., 2022; Hayden et al., 2023; Hamama, 2024). Constructs sitting between circumstance and outcome—acceptance, meaning-making, tolerance of ambivalence, benefit-finding—help explain divergence within similar family contexts, and are themselves patterned by the gendered and religious-cultural scripts discussed above.

Much existing research is grounded in Western individualistic contexts and may not generalize to collectivist societies (Mokoena and Kern, 2022; Long et al., 2022); in Latinx and certain Asian cultures, siblings may internalize the future caregiver role earlier and more naturally (Viswanathan et al., 2022; Long et al., 2022), which may amplify responsibility and future-oriented anxiety (Kisecik Sengul et al., 2026) while also providing a defined role and sense of purpose. More fundamentally, the core constructs of this field—burden, resilience, quality of life, even “over-involvement”—are themselves culturally situated. Instruments developed in one setting may not be measurement-invariant across others, and cross-cultural work needs to establish equivalence of measurement before comparing scores.

## 7 Gaps in the current literature and future research directions

Four gaps follow directly from the preceding analysis.

### 7.1 Populations

The overwhelming majority of studies have focused on school-aged and adolescent siblings, with comparatively little known about preschool-aged siblings, for whom the limited evidence—drawn from siblings of children with chronic disorders generally—remains inconclusive (Prentice et al., 2025). Research on adult siblings is also thin, particularly on future planning, relationship dynamics with adult autistic siblings, and the reciprocal exchange of emotional and practical support (Burke et al., 2024; Li et al., 2025). A nascent literature has begun to address emerging adulthood, acceptance, gender, and cultural context, so the priority now is consolidation and autism-specific extension rather than discovery.

### 7.2 Designs

Longitudinal studies of psychosocial adaptation are scarce; notably, prospective designs are well established in the infant-sibling tradition, where they have tracked siblings’ own social-communication development into the school years (Gangi et al., 2026), but this methodology has rarely been turned on adaptation in the sibling role. Reliance on parental report is a further constraint, since much of the family-level evidence summarized above is parent-reported (Koukouriki et al., 2021; Chang et al., 2024; Brinkman et al., 2023); future work should combine sibling self-report, teacher report, and direct observation within mixed-method designs (Brown et al., 2025), and a systematic review of sibling experience is currently in progress (Kantor et al., 2023). Population data-linkage of large sibling cohorts is a promising avenue for capturing long-term outcomes (Gray et al., 2025), provided that shared familial liability is modeled rather than ignored.

### 7.3 Mechanisms

Mechanistic understanding remains limited in three respects. First, it is not known how parental reflective functioning differs across children within the same family, or how such differences affect non-autistic siblings (Enav et al., 2026). Second, the pathways linking sibling bullying to mental health are only partly mapped: a longitudinal study identified self-esteem as a mediator among autistic adolescents (Deniz and Toseeb, 2023), but the corresponding pathway in non-autistic siblings has not been examined, and neither has the bidirectionality of sibling bullying. Third, the mechanisms by which cultural values moderate relations between stress and resilience remain to be specified (Long et al., 2022).

### 7.4 Comparators

Intervention studies have been conducted almost exclusively in autism populations, so whether sibling support needs are condition-specific or generic across chronic conditions remains unclear (Lynam and Smith, 2022).

## 8 Conclusion and future outlook

Several implications for clinical practice, family support, and policy can be drawn, and these differ in evidentiary strength. Assessing the family as an integrated unit, and maintaining routine attention to the psychosocial well-being of non-autistic siblings, are supported by convergent observational and population data (Lin et al., 2022; Bispo-Torres et al., 2023). That evidence documents elevated symptom rates rather than any benefit of screening, and no study has evaluated a sibling screening program; we therefore recommend routine attention within existing family contacts, with fuller assessment when risk indicators are present, rather than universal screening framed as case-finding. Age-appropriate psychoeducation about autism and channels for emotional expression are consistently valued by siblings themselves (Fjermestad and Lervik, 2025; Romney et al., 2025). Parents should be supported to communicate openly with non-autistic siblings, to validate their feelings and contributions, and to balance the allocation of family responsibilities, so that responsibility remains proportionate, acknowledged, and voluntary, particularly for older sisters (Trew, 2025b; Romney et al., 2025). Psychoeducational support groups and sibling skills training are feasible and acceptable, and one randomized trial supports improved relationship quality; pending larger controlled trials, they should be offered as promising rather than established practice (Zucker et al., 2022; Kaur et al., 2026). At the policy level, siblings should be recognized as stakeholders in their own right, and only conditionally as future support providers (Li et al., 2025; Long et al., 2025), and support programs should be explicitly incorporated into health and social care policies and funding frameworks to ensure accessibility and sustainability (Kaur et al., 2026).

Overall, the field has moved beyond asking whether sibling adaptation is good or bad. The more productive questions concern which siblings, in which family and cultural contexts, at which developmental points, benefit from which forms of support—and how much of what is currently described as sibling risk is attributable to siblinghood at all.
