# Supplementary material for: Psychosocial adaptation of siblings of autistic individuals: current evidence, controversies, and future directions
Source: Front Psychol. 2026 Sep 3;17:1858545. doi: 10.3389/fpsyg.2026.1858545 (PMC13583667; doi:10.3389/fpsyg.2026.1858545)
Supplement: Supplementary file 1 [file Supplementary_file_1.DOCX]

**Supplementary Material S1**

**Full search strategy**

This supplement reports the search strings used for each database. The search supported a structured but narrative synthesis; it was not prospectively registered, does not follow PRISMA guidance, and no formal risk-of-bias assessment or certainty grading was undertaken. Databases were searched from January 2021 to March 2026, and the reference lists of retrieved reviews were hand-searched for earlier landmark papers.

**Concept blocks**

Block 1 (population): sibling* OR brother* OR sister*

Block 2 (condition): autis* OR “autism spectrum disorder” OR ASD

Block 3 (outcome and intervention): psychosocial OR adjust* OR adapt* OR “mental health” OR “quality of life” OR resilien* OR intervention* OR support

The three blocks were combined with the Boolean operator AND.

**PubMed**

(“siblings”[MeSH Terms] OR sibling*[tiab] OR brother*[tiab] OR sister*[tiab]) AND (“autism spectrum disorder”[MeSH Terms] OR autis*[tiab] OR ASD[tiab]) AND (psychosocial[tiab] OR adjust*[tiab] OR adapt*[tiab] OR “mental health”[tiab] OR “quality of life”[tiab] OR resilien*[tiab] OR intervention*[tiab] OR support[tiab])

*Limits: publication date January 2021 to March 2026; English language.*

**PsycINFO**

(DE “Siblings” OR sibling* OR brother* OR sister*) AND (DE “Autism Spectrum Disorders” OR autis* OR ASD) AND (psychosocial OR adjust* OR adapt* OR “mental health” OR “quality of life” OR resilien* OR intervention* OR support)

*Limits: 2021 to 2026; English language; peer-reviewed journals.*

**Web of Science Core Collection**

TS=(sibling* OR brother* OR sister*) AND TS=(autis* OR “autism spectrum disorder” OR ASD) AND TS=(psychosocial OR adjust* OR adapt* OR “mental health” OR “quality of life” OR resilien* OR intervention* OR support)

*Refined by: publication years 2021 to 2026; document types Article or Review; English language.*

**Eligibility criteria**

Included: peer-reviewed empirical studies and reviews published in English reporting outcomes, experiences, or interventions concerning siblings of autistic individuals. Studies of siblings of individuals with intellectual and developmental disabilities, Down syndrome, or other chronic conditions were included only where autism-specific evidence was sparse, and are labelled as such throughout the main text and in Table 1.

Excluded: conference abstracts, dissertations, editorials, and studies reporting outcomes of the autistic individual alone, except where cited to illustrate bidirectional processes.

Screening and charting: titles and abstracts were screened by one author and checked by a second, with disagreements resolved by discussion. Included records were charted by population, developmental stage, study design, autism specificity, and principal finding.
